# Anxiety symptoms and associated factors in the Scleroderma Patient-centered Intervention Network (SPIN) cohort: a cross-sectional study

**DOI:** 10.1093/rheumatology/keaf374

**Published:** 2025-08-13

**Authors:** Sabrina Provencher, Marie-Eve Carrier, Gabrielle Virgili-Gervais, Meira Golberg, Richard S Henry, Linda Kwakkenbos, Catherine Fortuné, Amy Gietzen, Karen Gottesman, Geneviève Guillot, Amanda Lawrie-Jones, Maureen Sauvé, Susan J Bartlett, Laura K Hummers, Vanessa Malcarne, Maureen D Mayes, Michelle Richard, James Stempel, Robyn K Wojeck, Luc Mouthon, Andrea Benedetti, Brett D Thombs, Scleroderma Patient-centered Intervention Network Investigators

**Affiliations:** Lady Davis Institute for Medical Research, Jewish General Hospital, Montreal, QC, Canada; Department of Psychiatry, McGill University, Montreal, QC, Canada; Lady Davis Institute for Medical Research, Jewish General Hospital, Montreal, QC, Canada; Lady Davis Institute for Medical Research, Jewish General Hospital, Montreal, QC, Canada; Lady Davis Institute for Medical Research, Jewish General Hospital, Montreal, QC, Canada; Lady Davis Institute for Medical Research, Jewish General Hospital, Montreal, QC, Canada; Department of Psychiatry, McGill University, Montreal, QC, Canada; Department of Clinical Psychology, Behavioural Science Institute, Radboud University, Nijmegen, The Netherlands; Department of IQ Healthcare, Radboud University Medical Center, Nijmegen, The Netherlands; Centre for Mindfulness, Department of Psychiatry, Radboud University Medical Center, Nijmegen, The Netherlands; Ottawa Scleroderma Support Group, Ottawa, ON, Canada; National Scleroderma Foundation, Tri-State Chapter, Buffalo, NY, USA; National Scleroderma Foundation, Los Angeles, CA, USA; Sclérodermie Québec, Longueuil, QC, Canada; Scleroderma Australia and Scleroderma Victoria, Melbourne, Victoria, Australia; Scleroderma Society of Ontario and Scleroderma Canada, Hamilton, ON, Canada; Department of Medicine, McGill University, Montreal, QC, Canada; Research Institute of the McGill University Health Centre, Montreal, QC, Canada; Division of Rheumatology, Johns Hopkins University School of Medicine, Baltimore, MD, USA; Department of Psychology, San Diego State University, San Diego, CA, USA; San Diego Joint Doctoral Program in Clinical Psychology, San Diego State University and University of California, San Diego, CA, USA; Division of Rheumatology, University of Texas McGovern School of Medicine, Houston, TX, USA; Scleroderma Atlantic, Halifax, NS, Canada; Scleroderma Chicago, Chicago, IL, USA; Amgen Inc, Thousand Oaks, CA, USA; Service de Médecine Interne, Centre de Référence Maladies Autoimmunes et Autoinflammatoires Systémiques Rares d’Ile de France, de l’Est et de l’Ouest, Hôpital Cochin, Paris, France; Assistance Publique Hôpitaux de Paris-Centre, Hôpital Cochin, Université Paris Cité, Paris, France; Department of Medicine, McGill University, Montreal, QC, Canada; Department of Epidemiology, Biostatistics, and Occupational Health, McGill University, Montreal, QC, Canada; Respiratory Epidemiology and Clinical Research Unit, McGill University Health Centre, Montreal, QC, Canada; Lady Davis Institute for Medical Research, Jewish General Hospital, Montreal, QC, Canada; Department of Psychiatry, McGill University, Montreal, QC, Canada; Department of Medicine, McGill University, Montreal, QC, Canada; Department of Epidemiology, Biostatistics, and Occupational Health, McGill University, Montreal, QC, Canada; Department of Psychology, McGill University, Montreal, QC, Canada

**Keywords:** anxiety, PROMIS-29, scleroderma, SSc

## Abstract

**Objective:**

We (1) compared anxiety symptom levels in a multinational SSc cohort to a general population normative sample and (2) evaluated sociodemographic, lifestyle and SSc disease factors associated with symptoms.

**Methods:**

Scleroderma Patient-centered Intervention Network Cohort participants completed the Patient-Reported Outcomes Measurement Information System (PROMIS) Version 2 4a Anxiety domain upon enrolment. PROMIS domain scores use *T*-scores (mean = 50, S.D. = 10) calibrated to a United States normative sample. We compared *T*-scores to the PROMIS United States normative sample and, in SSc, assessed associations of sociodemographic, lifestyle and physician-reported disease-related variables with multivariable linear regression.

**Results:**

Among 2463 participants with SSc, mean anxiety symptom *T*-score (52.6, S.D. = 10.0, 95% CI 52.2, 53.0) was ∼1/3 S.D. higher than the United States general population mean of 50 (S.D. = 10), though within normal limits. Higher *T*-scores were associated with younger age (1.07 *T*-score points per 10 years, 95% CI 0.74, 1.40), female sex (1.81, 95% CI 0.63, 3.00), non-married status (0.99, 95% CI 0.14, 1.84), race or ethnicity other than White (1.79, 95% CI 0.72, 2.85), living in Canada (1.70, 95% CI 0.61, 2.79), the United Kingdom (1.53, 95% CI 0.06, 2.99) or France (2.01, 95% CI 0.98, 3.03) (vs the United States), higher BMI (0.11, 95% CI 0.03, 0.17), less time since non-Raynaud’s symptom onset (0.82 per 10 years, 95% CI 0.40, 1.30), gastrointestinal involvement (2.70, 95% CI 1.52, 3.88), moderate small joint contractures (1.24, 95% CI 0.10, 2.38), the absence of interstitial lung disease (0.93, 95% CI −1.79, −0.07) and Sjögren disease (1.67, 95% CI 0.17, 3.17). Interstitial lung disease was not statistically significant when accounting for an interaction with country. Anxiety was also associated with pruritus and pain intensity in a sensitivity analysis that included variables with possible bi-directional pathways with anxiety.

**Conclusion:**

Anxiety symptoms were somewhat elevated among individuals with SSc and associated with multiple sociodemographic and disease factors.

Rheumatology key messages

- We evaluated factors associated with anxiety symptoms in over 2400 people with SSc.
- Levels of anxiety symptoms were somewhat higher in SSc than in a general population sample.
- Multiple sociodemographic and SSc disease factors were statistically significantly associated with higher anxiety symptoms.

## Introduction

SSc is a rare autoimmune connective tissue disease characterized by skin fibrosis and pathological changes to internal organs including the lungs, kidneys, gastrointestinal tract and heart [1]. Significant challenges include fatigue, pain, sleep difficulty, body image distress from appearance changes, fear of disease progression and mental health challenges [2–4].

Anxiety is more common in people with chronic diseases compared to the general population [5] and is associated with female sex [6, 7]; younger age [5–8]; low socioeconomic status [7, 8]; being disabled or unemployed [7]; being separated, divorced or widowed [8]; non-Hispanic or Black race or ethnicity [8]; and smoking [8]. In chronic diseases, risk factors include number of medical conditions [8], more severe disease, and treatment nonadherence [7].

People with SSc may experience anxiety due to its unpredictable course and fears of disease progression [4] or because of disfiguring appearance changes and body image concerns [9]. Anxiety prevalence in SSc, however, is uncertain. A 2023 systematic review identified only two studies that evaluated anxiety disorder prevalence [10]. Point prevalence of any anxiety disorder was 50% in 100 French patient conference attendees (*N* = 100) and inpatients (*N* = 100), including 13% with social anxiety disorder and 13% with generalized anxiety disorder [11]. Among 93 outpatients from India, however, only 3% met generalized anxiety disorder criteria [12].

Based on an ongoing living systematic review [10], updated to 1 April 2025 [13], only one study (*N* = 114) has examined factors associated with anxiety. That study did not find any associations, but confidence in conclusions was limited by the small sample, study design aspects and the small set of variables analysed.

We (1) compared anxiety symptoms in SSc to general population norms, and (2) identified associated sociodemographic, lifestyle and disease factors.

## Materials and methods

This cross-sectional study used data collected at enrolment of Scleroderma Patient-centered Intervention Network (SPIN) Cohort participants [14, 15]. A protocol was posted online (https://osf.io/2uxmk/) prior to study initiation. The study was reported consistent with STROBE [16] guidance. Methods from SPIN Cohort studies are similar. Thus, we followed reporting guidance from the Text Recycling Research Project [17].

### Participants and procedures

The SPIN Cohort is a convenience sample of participants from 47 sites in seven countries (Australia, Canada, France, Mexico, Spain, United Kingdom, United States). Eligible participants are invited by site personnel to enrol during regular visits. Eligible SPIN Cohort participants are aged ≥18 years; fluent in English, French or Spanish; and classified as having SSc based on 2013 American College of Rheumatology/European League Against Rheumatism criteria [18]. Written informed consent is obtained, the recruiting site physician or nurse coordinator submits an online medical data form, and participants receive an email to complete their baseline assessment and, subsequently, assessments at 3-month intervals. We included baseline data from inception (April 2014) through April 2024. The SPIN Cohort was approved by the Research Ethics Committee of the Centre intégré universitaire de santé et de services sociaux du Centre-Ouest-de-l’Île-de-Montréal (#MP-05–2013-150) and the ethics committees of recruiting sites.

### Measures

Participants provided sociodemographic variables (race or ethnicity, education, marital status) and lifestyle information (smoking status, alcohol consumption). Physicians reported age, sex, height, weight, years since onset of non-Raynaud’s phenomenon symptoms, SSc subtype (limited cutaneous, diffuse cutaneous, sine), modified Rodnan Skin Score (mRSS), gastrointestinal symptoms (upper, lower, no gastrointestinal involvement), digital ulcers anywhere on the fingers, tendon friction rubs (currently, in the past, never), small or large joint contractures (none, mild [≤25% range of motion limitation], moderate to severe [>25%]), pulmonary arterial hypertension, interstitial lung disease, history of scleroderma renal crisis, overlap syndromes (SLE, RA, Sjögren disease, autoimmune thyroid disease, idiopathic inflammatory myopathy, primary biliary cholangitis) and SSc-related antibodies (antinuclear antibody, anti-centromere, anti-topoisomerase I and anti-RNA polymerase III). See Supplementary Data S1 for data collection forms.

#### Anxiety symptoms

Anxiety symptoms were evaluated using the 4a Short Form of the Patient-Reported Outcomes Information System version 2.0 (PROMIS-29v2.0) 4-item Anxiety domain [19]. Items assess symptoms in the last 7 days and are scored on a 5-point Likert scale (1 = ‘Never’; 5 = ‘Always’). The total score is converted into a *T*-score (mean = 50, S.D. = 10), which was calibrated using a United States general population sample (*N* = 788) [20] that reflected 2000 United States Census demographics [21]. Higher scores represent greater anxiety. A normal symptom level is represented by a *T*-score <55.0, mild symptoms 55.0 to 59.9, moderate symptoms 60.0 to 70.0 and severe symptoms >70.0 [22]. The PROMIS-29v2.0 has been validated in the SPIN Cohort [23]. Minimal important change estimates are from 2.3 to 3.5 *T*-score points [24].

#### Pruritus

Pruritus severity was evaluated with a single item, ‘In the past week, how severe was your itch?’ (numerical rating scale; 0 = not severe at all, 10 = unbearable), which is valid for assessing pruritus severity [25].

#### Pain

Pain intensity in the last week was assessed with the PROMIS-29v2.0 (numerical rating scale; 0 = no pain, 10 = worst imaginable pain) [26, 27]. Single-item pain measurements perform equivalently to multi-item scales in SSc [28]. Pain interference in the last week was assessed with the 4-item PROMIS-29v2.0 domain (5-point Likert scale; 1 = ‘Not at all’, 5 = ‘Very much’) with total score calibrated to the United States general population (mean = 50, S.D. = 10) and higher scores representing greater pain interference.

### Statistical analysis

Associations of sociodemographic, lifestyle and disease-related variables with anxiety symptoms were assessed using multivariable linear regression and logistic regression (outcome = moderate or severe symptom levels). We identified model variables *a priori* based on studies of factors in the general population and chronic diseases [3–8] and the experience of research team members with SSc. We did not include psychosocial or functional variables (pruritus, pain, fatigue) as predictors in the main model because they are also outcomes of SSc disease manifestations and likely have bi-directional causal associations with anxiety symptoms. We did this to avoid reverse causality, by which outcome variables may be causally associated with predictor variables, which leads to (1) biased model coefficients, potentially masking associations of model variables with anxiety symptoms; (2) spuriously inflated goodness-of-fit estimates (*R*^2^); and (3) the inability to assess potential causal influences between variables for which reverse causation is likely [29].

Main analysis variables were age (years); sex (male vs female [reference]); education (years); marital status (single or divorced/separated/widowed vs married or living as married [reference]); self-reported race or ethnicity (non-White vs White [reference]); country (Canada, France, United Kingdom, other [Australia, Mexico, Spain] vs United States [reference]); BMI (continuous); time since first non-Raynaud’s symptom (years); disease subtype (diffuse SSc vs limited SSc (including sine) [reference]); gastrointestinal involvement (upper gastrointestinal involvement (esophagus) or lower gastrointestinal involvement (stomach or intestine) vs no gastrointestinal involvement [reference]); digital ulcers (anywhere on finger vs none [reference]); current tendon friction rubs (present, present in the past vs never present [reference]); small joint contractures (moderate, severe vs none or mild [reference]); large joint contractures (moderate, severe vs none or mild [reference]); history of scleroderma renal crisis (yes, no [reference]); interstitial lung disease (yes, no [reference]); pulmonary arterial hypertension (yes, no [reference]); overlap syndromes (SLE (yes, no [reference]); RA (yes, no [reference]); Sjögren disease (yes, no [reference]); autoimmune thyroid disease (yes, no [reference]); idiopathic inflammatory myopathy (yes, no [reference]); primary biliary cholangitis (yes, no [reference])). See Supplementary Table S1.

We accounted for missing data with multiple imputations via chained equations, using the mice and bootImpute packages in R [30]. We imputed two datasets using the mice package for each of 200 bootstrapped samples and computed their quantiles [31, 32]. This enabled us to generate 95% CIs that reflect uncertainty induced by bootstrapping and imputation data. Variables included all main regression model and sensitivity analysis variables, including the anxiety outcome, plus alcohol consumption, smoking status and PROMIS-29v2 depression symptom, fatigue, sleep and physical function domain scores.

We conducted four multivariable sensitivity analyses. We (1) conducted a complete case analysis; (2) added pruritus and pain intensity to the main model; (3) replaced disease subtype with continuous mRSS; and (4) added SSc-related antibodies to the main model. We included pruritus and pain as a sensitivity analysis because of possible bi-directional causal associations with anxiety.

Additionally, we found a large association of anxiety symptoms with country and, unexpectedly, a negative association with interstitial lung disease. Thus, *post hoc*, we evaluated all model variables to assess whether interactions with country may have influenced model estimates.

For all models, we reported unstandardized regression coefficients with 95% CIs. We assessed multicollinearity among variables in all models by calculating generalized variance inflation factors (GVIF) and condition indices. GVIF ≥5 and condition indices ≥10 suggest multicollinearity may be a concern [33]. All regression analyses were conducted in R (R version 4.3.1, RStudio Version 2024.4.2.764).

## Results

We included 2463 participants who were predominantly female (*N* = 2146; 87%) and White (*N* = 2032; 83%). Mean age was 54.9 (S.D. = 12.7) years. Mean education was 15.0 (S.D. = 3.7) years. Most were from the United States (*n* = 835; 34%), France (*n* = 761; 31%) or Canada (*n* = 526; 21%). Mean time since non-Raynaud’s symptom onset was 10.8 years (S.D. = 8.8 years), and 957 (39%) participants had diffuse cutaneous SSc. Table 1 shows baseline characteristics, including the number of participants with data for each variable.

**Table 1.** Sample sociodemographic and disease characteristics

	Full sample (*N* = 2463)		Limited cutaneous SSc^a^ (*N* = 1506)		Diffuse cutaneous SSc (*N* = 957)		Female (*N* = 2146)		Male (*N* = 317)	
	*N* ^b^	Mean (S.D.) or *N* (%)	*N*	Mean (S.D.) or *N* (%)	*N*	Mean (S.D.) or *N* (%)	*N*	Mean (S.D.) or *N* (%)	*N*	Mean (S.D.) or *N* (%)
Age (years)	2463	54.9 (12.7)	1506	56.6 (12.5)	957	52.2 (12.5)	2146	54.5 (12.8)	317	57.4 (11.8)
Sex	2463		1506		957		2146		317	
Female		2146 (87%)		1343 (89%)		803 (84%)		2146 (100%)		0 (0%)
Male		317 (13%)		163 (11%)		154 (16%)		0 (0%)		317 (100%)
Education (years)	2453	15.0 (3.7)	1498	14.9 (3.9)	955	15.1 (3.5)	2138	15.0 (3.7)	315	14.4 (4.0)
Marital status	2458		1502		956		2141		317	
Married or living as married		1707 (69%)		1063 (71%)		644 (67%)		1453 (68%)		254 (80%)
Single, divorced/separated, widowed		751 (31%)		439 (29%)		312 (33%)		688 (32%)		63 (20%)
Race or ethnicity^c^	2456		1502		954		2139		317	
White		2032 (83%)		1306 (87%)		726 (76%)		1763 (82%)		269 (85%)
Non-white		424 (17%)		196 (13%)		228 (24%)		376 (18%)		48 (15%)
Country	2463		1506		957		2146		317	
United States		835 (34%)		444 (29%)		391 (41%)		724 (34%)		111 (35%)
France		761 (31%)		505 (34%)		256 (27%)		647 (30%)		114 (36%)
Canada		526 (21%)		340 (23%)		186 (19%)		463 (22%)		63 (20%)
United Kingdom		241 (10%)		144 (10%)		97 (10%)		219 (10%)		22 (7%)
Australia, Mexico, Spain		100 (4%)		73 (5%)		27 (3%)		93 (4%)		7 (2%)
Smoking status	2459		1503		956		2142		317	
Smoker		184 (7%)		121 (8%)		63 (7%)		160 (7%)		24 (8%)
Non-smoker		2275 (93%)		1382 (92%)		893 (93%)		1982 (93%)		293 (92%)
Alcohol consumption (drinks per week)	2454	2.0 (4.0)	1501	2.1 (4.3)	953	1.8 (3.5)	2139	1.8 (3.6)	315	3.4 (6.0)
BMI	2463	25.3 (5.6)	1506	25.5 (5.6)	957	24.9 (5.7)	2146	25.2 (5.8)	317	25.8 (4.2)
Years since first non-Raynaud’s symptoms	2265	10.8 (8.8)	1375	12.1 (9.3)	890	9.0 (7.5)	1967	11.1 (8.9)	298	9.4 (7.9)
Disease subtype	2463		1506		957		2146		317	
Diffuse		957 (39%)		0 (0%)		957 (100%)		803 (37%)		154 (49%)
Limited or sine^a^		1506 (61%)		1506 (100%)		0 (0%)		1343 (63%)		163 (51%)
mRSS	2054	7.6 (8.0)	1259	4.2 (4.1)	795	13.1 (9.4)	1785	7.4 (7.9)	269	9.0 (8.6)
Gastrointestinal involvement	2442		1499		943		2126		316	
Yes		2130 (87%)		1294 (86%)		836 (89%)		1861 (88%)		269 (85%)
No		312 (13%)		205 (14%)		107 (11%)		265 (12%)		47 (15%)
Digital ulcers	2362		1457		905		2065		297	
Yes		374 (16%)		139 (10%)		235 (26%)		316 (15%)		58 (20%)
No		1988 (84%)		1318 (90%)		670 (74%)		1749 (85%)		239 (80%)
Tendon friction rubs	2172		1356		816		1887		285	
Current		232 (11%)		1193 (88%)		112 (14%)		194 (10%)		38 (13%)
Past		229 (11%)		120 (9%)		186 (23%)		187 (10%)		42 (15%)
Never		1711 (79%)		43 (3%)		518 (63%)		1506 (80%)		205 (72%)
Small joint contractures	2329		1434		895		2026		303	
None or mild		1713 (74%)		1211 (84%)		502 (56%)		1506 (74%)		207 (68%)
Moderate		442 (19%)		169 (12%)		273 (31%)		370 (18%)		72 (24%)
Severe		174 (7%)		54 (4%)		120 (13%)		150 (7%)		24 (8%)
Large joint contractures	2286		1407		879		1990		296	
None or mild		2002 (88%)		1306 (93%)		696 (79%)		1746 (88%)		256 (86%)
Moderate		210 (9%)		70 (5%)		140 (16%)		177 (9%)		33 (11%)
Severe		74 (3%)		31 (2%)		43 (5%)		67 (3%)		7 (2%)
History of scleroderma renal crisis	2428		1491		937		2114		314	
Yes		104 (4%)		24 (2%)		80 (9%)		87 (4%)		17 (5%)
No		2324 (96%)		1467 (98%)		857 (91%)		2027 (96%)		297 (95%)
Interstitial lung disease	2413		1481		932		2101		312	
Yes		851 (35%)		401 (27%)		450 (48%)		686 (33%)		165 (53%)
No		1562 (65%)		1080 (73%)		482 (52%)		1415 (67%)		147 (47%)
Pulmonary arterial hypertension	2346		1445		901		2047		299	
Yes		211 (9%)		134 (9%)		77 (9%)		176 (9%)		35 (12%)
No		2135 (91%)		1311 (91%)		824 (91%)		1871 (91%)		264 (88%)
Pruritus	2154	1.8 (2.6)	1300	1.6 (2.5)	854	2.1 (2.8)	1885	1.8 (2.7)	269	1.5 (2.3)
Pain intensity	2460	3.6 (2.6)	1503	3.5 (2.6)	957	3.9 (2.6)	2143	3.7 (2.6)	317	3.2 (2.4)
Pain interference	2463	55.6 (9.7)	1506	54.8 (9.6)	957	56.7 (9.7)	2146	55.7 (9.7)	317	54.5 (9.2)
SLE	2401		1478		923		2094		307	
Yes		67 (3%)		44 (3%)		23 (2%)		61 (3%)		6 (2%)
No		2334 (97%)		1434 (97%)		900 (98%)		2033 (97%)		301 (98%)
RA	2400		1476		924		2093		307	
Yes		134 (6%)		69 (5%)		65 (7%)		123 (6%)		11 (4%)
No		2266 (94%)		1407 (95%)		859 (93%)		1970 (94%)		296 (96%)
Sjögren disease	2362		1453		909		2058		304	
Yes		181 (8%)		128 (9%)		53 (6%)		168 (8%)		13 (4%)
No		2181 (92%)		1325 (91%)		856 (94%)		1890 (92%)		291 (96%)
Autoimmune thyroid disease	2355		1447		908		2053		302	
Yes		146 (6%)		101 (7%)		45 (5%)		139 (7%)		7 (2%)
No		2209 (94%)		1346 (93%)		863 (95%)		1914 (93%)		295 (98%)
Idiopathic inflammatory myopathy	2399		1480		919		2095		304	
Yes		125 (5%)		63 (4%)		62 (7%)		104 (5%)		21 (7%)
No		2274 (95%)		1417 (96%)		857 (93%)		1991 (95%)		283 (93%)
Primary biliary cholangitis	2378		1463		915		2076		302	
Yes		45 (2%)		38 (3%)		7 (1%)		43 (2%)		2 (1%)
No		2333 (98%)		1425 (97%)		908 (99%)		2033 (98%)		300 (99%)
Antinuclear antibodies	2271		1410		861		1977		294	
Positive		2146 (94%)		1347 (96%)		799 (93%)		1872 (95%)		274 (93%)
Negative		125 (6%)		63 (4%)		62 (7%)		105 (5%)		20 (7%)
Anti-centromere	1937		1220		717		1690		247	
Positive		694 (36%)		637 (52%)		57 (8%)		1047 (62%)		51 (21%)
Negative		1243 (64%)		583 (48%)		660 (92%)		643 (38%)		196 (79%)
Anti-topoisomerase I [Scl70]	2154		1317		837		1870		284	
Positive		577 (27%)		252 (19%)		325 (39%)		494 (26%)		83 (29%)
Negative		1577 (73%)		1065 (81%)		512 (61%)		1376 (74%)		201 (71%)
Anti-RNA polymerase III	1420		850		570		1226		194	
Positive		255 (18%)		51 (6%)		204 (36%)		219 (18%)		36 (19%)
Negative		1165 (82%)		799 (94%)		366 (64%)		1007 (82%)		158 (81%)

mRSS = modified Rodnan Skin Score.

a Includes 73 participants with sine SSc.

b *N* for some variables <2463 due to missing data.

c Race or ethnicity data were self-reported in each country using standard categories used in that country. Therefore, categories differed between countries.

Table 2 shows PROMIS-29v2.0 anxiety symptom score means and number and percentage in each severity level category overall and by country, disease subtype, and sex. Mean anxiety symptom score was 52.6 (S.D. = 10.0; 95% CI 52.2 to 53.0), which is higher than the United States general population mean of 50 (S.D. = 10), though within normal limits. There were 1350 (55%) participants with scores within normal limits; 550 (22%) with mildly elevated scores; 460 (19%) with moderately elevated scores; and 103 (4%) with severely elevated scores.

**Table 2.** Anxiety symptoms by country, disease subtype, and sex

	Full sample (*N* = 2463)^a^	Country					Disease subtype		Sex	
		US (*N* = 835)	France (*N* = 761)	Canada (*N* = 526)	UK (*N* = 241)	Other^b^ (*N* = 100)	Limited cutaneous or sine (*N* = 1506)	Diffuse cutaneous (*N* = 957)	Female (*N* = 2146)	Male (*N* = 317)
Anxiety symptoms, *T*-score mean	52.6 (10.0)	51.4 (9.9)	53.6 (10.1)	52.7 (10.0)	53.5 (10.6)	51.4 (8.4)	52.1 (10.0)	53.3 (10.0)	52.9 (10.1)	50.7 (9.8)
Anxiety symptoms										
Severe (*T*-score >70)	103 (4%)	26 (3%)	44 (6%)	22 (4%)	10 (4%)	1 (1%)	57 (4%)	46 (5%)	92 (4%)	11 (3%)
Moderate (*T*-score 60–70)	460 (19%)	131 (16%)	166 (22%)	92 (18%)	58 (24%)	13 (13%)	270 (18%)	190 (20%)	417 (19%)	43 (14%)
Mild (*T*-score 55–59.9)	550 (22%)	181 (22%)	160 (21%)	125 (24%)	58 (24%)	26 (26%)	332 (22%)	218 (23%)	485 (23%)	65 (21%)
Within normal limits (*T*-score <55)	1,350 (55%)	497 (60%)	391 (51%)	287 (55%)	115 (48%)	60 (60%)	847 (56%)	503 (53%)	1,152 (54%)	198 (62%)

a Thirty participants (1% of total) completed baseline measures in March to May 2020 during a period when we previously reported that symptoms were higher than normal among SPIN Cohort participants; symptoms returned to pre-pandemic levels by the end of May 2020 [34, 35].

b Includes 39 participants from Australia, 21 from Mexico and 40 from Spain.

Table 3 shows results from bivariate and multivariable linear regressions (outcome *T*-score points). In multivariable analysis, younger age (0.11 points per year, 95% CI 0.07 to 0.14; 1.06 points per 10 years, 95% CI 0.74 to 1.40); female sex (1.81 points, 95% CI −3.00 to −0.63); being single, divorced, separated or widowed (0.99 points, 95% CI 0.14 to 1.84); self-reported race or ethnicity other than White (1.79 points, 95% CI 0.72 to 2.85); and living in Canada (1.70 points, 95% CI 0.61 to 2.79), the United Kingdom (1.53 points, 95% CI 0.06 to 2.99) or France (2.01 points, 95% CI 0.98 to 3.03), vs the United States, were independently associated with greater anxiety symptoms. Higher BMI (0.10 points per BMI point, 95% CI 0.03 to 0.17) was also statistically significantly associated with higher anxiety symptom scores. Among SSc-related variables, there were statistically significant positive associations with shorter time since non-Raynaud’s symptom onset (0.08 points per S.D., 95% CI 0.04 to 0.13; 0.82 points per 10 years, 95% CI 0.35 to 1.29), gastrointestinal involvement (2.70 points, 95% CI 1.52 to 3.88), moderate small joint contractures (1.24 points, 95% CI 0.10 to 2.38) and Sjögren disease overlap (1.67 points, 95% CI 0.17 to 3.17). Variables not statistically significantly associated with anxiety symptoms in multivariable analysis were education; living in Australia, Mexico or Spain; disease subtype, digital ulcers; current or past tendon friction rubs; severe small joint contractures; moderate or severe large joint contractures; history of scleroderma renal crisis; pulmonary arterial hypertension; SLE; RA; autoimmune thyroid disease; idiopathic inflammatory myopathy; and primary biliary cholangitis.

**Table 3.** Linear regression analysis of sociodemographic and disease characteristic associations with anxiety symptoms

	Anxiety symptoms	
	Full sample (*N* = 2463)	
	Bivariate regression coefficient (95% CI)^a^	Multivariable regression coefficient (95% CI)^a^
Sociodemographic variables and BMI		
Age (years)	**−0.12 (−0.16; −0.09)**	**−0.11 (−0.14; −0.07)**
Male sex (reference = female)	**−2.17 (−3.35; −0.99)**	**−1.81 (−3.00; −0.63)**
Years of education (years)	−0.07 (−0.18; 0.03)	−0.09 (−0.20; 0.01)
Single, divorced/separated or widowed (reference = married or living as married)	**1.65 (0.79; 2.50)**	**0.99 (0.14; 1.84)**
Non-White (reference = White)	**2.52 (1.47; 3.56)**	**1.79 (0.72; 2.85)**
Country (reference = United States)		
Canada	**1.29 (0.20; 2.38)**	**1.70 (0.61; 2.79)**
United Kingdom	**2.09 (0.65; 3.52)**	**1.53 (0.06; 2.99)**
France	**2.21 (1.23; 3.19)**	**2.01 (0.98; 3.03)**
Other (Australia, Mexico, Spain)	−0.06 (−2.13; 2.02)	0.03 (−2.02; 2.09)
BMI	0.06 (−0.01; 0.13)	**0.10 (0.03; 0.17)**
Disease variables		
Years since first non-Raynaud’s symptoms (years)	**−0.09 (−0.14; −0.05)**	**−0.08 (−0.13; −0.04)**
Diffuse subtype (reference = limited or sine)	**1.04 (0.22; 1.85)**	0.07 (−0.86; 1.00)
Gastrointestinal involvement (reference = no)	**2.51 (1.32; 3.69)**	**2.70 (1.52; 3.88)**
Digital ulcers (reference = no)	**1.43 (0.35; 2.50)**	0.91 (−0.24; 2.05)
Tendon friction rubs (reference = never)		
Current	1.15 (−0.12; 2.43)	0.19 (−1.11; 1.49)
Past	0.37 (−0.91; 1.65)	−0.07 (−1.41; 1.28)
Small joint contractures (reference = none or mild)		
Moderate	**1.51 (0.49; 2.53)**	**1.24 (0.10; 2.38)**
Severe	**1.63 (0.13; 3.12)**	0.83 (−0.87; 2.54)
Large joint contractures (reference = none or mild)		
Moderate	**1.61 (0.26; 2.96)**	−0.02 (−1.51; 1.46)
Severe	**2.35 (0.15; 4.55)**	1.83 (−0.53; 4.19)
History of scleroderma renal crisis (reference = no)	1.27 (−0.66; 3.21)	0.81 (−1.14; 2.75)
Interstitial lung disease (reference = no)	−0.47 (−1.30; 0.36)	**−0.93 (−1.79; −0.07)**
Pulmonary arterial hypertension (reference = no)	−0.67 (−2.08; 0.73)	0.16 (−1.26; 1.57)
Overlap syndromes		
SLE (reference = no)	0.46 (−1.94; 2.87)	−0.69 (−3.08; 1.70)
RA (reference = no)	1.11 (−0.60; 2.83)	0.90 (−0.84; 2.63)
Sjögren disease (reference = no)	**1.53 (0.04; 3.01)**	**1.67 (0.17; 3.17)**
Autoimmune thyroid disease (reference = no)	0.13 (−1.50; 1.76)	−0.62 (−2.22; 0.98)
Idiopathic inflammatory myopathy (reference = no)	0.57 (−1.22; 2.36)	−0.43 (−2.21; 1.36)
Primary biliary cholangitis (reference = no)	1.74 (−1.16; 4.63)	2.38 (−0.48; 5.23)

Adjusted *R*^2^ = 0.06.

a All regression coefficients are unstandardized. Bolded results are statistically significant.

The absence of interstitial lung disease (0.93 points, 95% CI −1.79 to −0.07) was unexpectedly associated with higher anxiety symptom scores. We identified a statistically significant interaction between interstitial lung disease and country, however (Supplementary Table S2). Interaction terms were negative and large for Canada, the United Kingdom, and France, all compared with the United States (only France statistically significant), suggesting that the association of having interstitial lung disease and anxiety symptoms may be more positive in the United States than other countries. In this model, which included the interaction term, interstitial lung disease was no longer associated with anxiety symptoms (0.21 points, 95% CI −1.17 to 1.59). No other model variables changed substantively from the main analysis.

No variables included in the main analysis had GVIF ≥ 5. Autoimmune thyroid disease, idiopathic inflammatory myopathy, and primary biliary cholangitis had condition indices above 10, indicating possible multicollinearity. However, when these variables were removed from the model, the magnitude and statistical significance of other variables did not change.

As shown in Table 4, results from multivariable logistic regression (outcome moderate or severe anxiety symptoms) were similar to those from linear multivariable regression. Complete case analysis results (*N* = 1736) were similar to main analysis results (see Supplementary Table S3). In the sensitivity analysis that included pruritus and pain intensity, both pruritus (0.27 points per point on a 0 to 10 numerical rating scale, 95% CI 0.12 to 0.42); and pain intensity (1.41 points per point on a 0 to 10 numerical rating scale, 95% CI 1.25 to 1.57) were associated with higher anxiety symptoms (Supplementary Table S4). Other sensitivity analyses found that mRSS and SSc-related antibodies were not associated with anxiety symptoms (Supplementary Tables S5 and S6).

**Table 4.** Logistic regression analysis of sociodemographic and disease characteristic associations with binary anxiety symptoms

	Binary anxiety symptoms	
	Full sample (*N* = 2463)	
	Bivariate regression Unadjusted odds ratios (95% CI)^a^	Logistic regression Adjusted odds ratios (95% CI)^a^
Sociodemographic variables and BMI		
Age (years)	**0.98 (0.97; 0.98)**	**0.98 (0.97; 0.99)**
Male sex (reference = female)	**0.66 (0.48; 0.89)**	**0.67 (0.48; 0.92)**
Years of education (years)	0.98 (0.96; 1.01)	0.98 (0.95; 1.00)
Single, divorced/separated or widowed (reference = married or living as married)	**1.30 (1.07; 1.59)**	1.17 (0.95; 1.44)
Non-White (reference = White)	**1.52 (1.20; 1.92)**	**1.34 (1.04; 1.73)**
Country (reference = United States)		
Canada	1.20 (0.91; 1.56)	1.32 (1.00; 1.76)
United Kingdom	**1.70 (1.22; 2.35)**	**1.61 (1.12; 2.29)**
France	**1.65 (1.30; 2.08)**	**1.67 (1.29; 2.16)**
Other (Australia, Mexico, Spain)	0.70 (0.37; 1.23)	0.73 (0.38; 1.31)
BMI	1.01 (1.00; 1.03)	1.02 (1.00; 1.04)
Disease variables		
Years since first non-Raynaud’s symptoms (years)	**0.97 (0.96; 0.99)**	**0.98 (0.96; 0.99)**
Diffuse subtype (reference = limited or sine)	1.15 (0.95; 1.40)	0.93 (0.73; 1.17)
Gastrointestinal involvement (reference = no)	**1.53 (1.13; 2.11)**	**1.65 (1.20; 2.30)**
Digital ulcers (reference = no)	1.22 (0.95; 1.56)	1.17 (0.88; 1.55)
Tendon friction rubs (reference = never)		
Current	**1.34 (1.00; 1.78)**	1.13 (0.82; 1.54)
Past	1.12 (0.83; 1.50)	1.10 (0.79; 1.54)
Small joint contractures (reference = none or mild)		
Moderate	1.26 (0.99; 1.59)	1.17 (0.88; 1.55)
Severe	1.13 (0.79; 1.59)	0.88 (0.57; 1.35)
Large joint contractures (reference = none or mild)		
Moderate	1.32 (0.96; 1.77)	1.01 (0.70; 1.44)
Severe	1.61 (0.99; 2.56)	1.65 (0.94; 2.83)
History of scleroderma renal crisis (reference = no)	1.38 (0.89; 2.10)	1.36 (0.85; 2.13)
Interstitial lung disease (reference = no)	0.88 (0.72; 1.07)	0.82 (0.66; 1.02)
Pulmonary arterial hypertension (reference = no)	0.86 (0.60; 1.20)	1.06 (0.73; 1.52)
Overlap syndromes		
SLE (reference = no)	1.02 (0.56; 1.76)	0.85 (0.46; 1.51)
RA (reference = no)	1.33 (0.90; 1.93)	1.34 (0.88; 2.02)
Sjögren disease (reference = no)	1.30 (0.92; 1.80)	1.38 (0.96; 1.97)
Autoimmune thyroid disease (reference = no)	0.73 (0.47; 1.09)	**0.60 (0.38; 0.92)**
Idiopathic inflammatory myopathy (reference = no)	1.13 (0.74; 1.69)	0.93 (0.59; 1.43)
Primary biliary cholangitis (reference = no)	0.91 (0.43; 1.77)	1.06 (0.48; 2.13)

a Bolded results are statistically significant.

## Discussion

Among 2463 participants with SSc from seven countries, the mean *T*-score for anxiety symptoms was 52.6, which is almost 0.3 S.D. greater than the US general population mean. There were 1113 (45%) participants who reported mild, moderate, or severe anxiety symptoms in the past 7 days, including 563 (23%) with moderate or severe symptoms. Younger age; female sex; being single, divorced, separated or widowed; self-reported race or ethnicity other than White; living in Canada, the United Kingdoms or France; and higher BMI were associated with higher anxiety symptom scores. Among disease variables, years since onset of non-Raynaud’s symptoms, gastrointestinal involvement, the presence of moderate small joint contractures, the absence of interstitial lung disease, and the presence of overlap syndromes including Sjögren disease were associated with greater anxiety symptoms. The initial finding that interstitial lung disease was associated with lower anxiety symptom scores appears to reflect an unaccounted-for interaction. Interstitial lung disease was much more strongly associated with anxiety symptoms among participants from the United States than other countries. When we included a country × interstitial lung disease interaction term in the model, interstitial lung disease was no longer associated with anxiety symptoms.

A living systematic review of mood and anxiety disorders and symptoms in SSc [10, 13] identified two studies that have evaluated the prevalence of anxiety disorders in SSc. One study reported that current prevalence of any anxiety disorder was 49% (95% CI 12%, 27%) for 51 French conference attendees and 51% (95% CI 38%, 64%) for 49 French inpatients. The other reported that current generalized anxiety disorder prevalence was 3% for outpatients in India (95% CI 1%, 9%; *N* = 93) [10]. Our study did not assess anxiety disorders, but we found somewhat higher levels of anxiety symptom scores among SSc patients compared with the United States general population based on the anxiety domain of the PROMIS-29 v2.0 scale [19]. Based on estimates of minimal important change (2.3 to 3.5 points), the 2.6 *T*-score point difference we found appears to be potentially clinically meaningful, although there is uncertainty about existing minimal important change estimates [24].

We previously studied levels of anxiety symptoms in SPIN Cohort participants prior to and during the COVID-19 pandemic [34, 35]. We found that anxiety levels among the 435 participants in the study, based on the Anxiety domain of the PROMIS-29 v2.0 scale, initially increased substantially in April 2020 (standardized mean difference = 0.51, 95% CI 0.37 to 0.64) but returned to pre-pandemic levels by the end of May 2020 and remained at pre-pandemic levels thereafter. Only 30 SPIN Cohort participants (1% of total in the present study) enrolled and completed baseline cohort measures during March to May 2020; thus, this would not have influenced symptom level estimates or factors associated with anxiety in our analyses.

The living systematic review on mental health in SSc [10] has included only one study that assessed factors associated with anxiety symptoms, however the review authors concluded that they could not be confident in the factors identified or not identified in that study given limitations in the study design, the small number of variables assessed, and the small sample size for multivariable regression (*N* = 114). In the present study, sociodemographic variables, including female sex and self-reported race or ethnicity other than White were robustly associated with anxiety symptoms, which is consistent with general population studies [8]. Among disease manifestations, gastrointestinal involvement was most strongly associated with anxiety symptoms, which is consistent with previous findings on factors associated with depression symptoms in SSc [3]. This may reflect the major influence of gastrointestinal symptoms on quality of life in SSc [36].

The adjusted *R*^2^ for the main multivariable model was 0.06, which may appear low. Models examining people with chronic conditions typically explain a small amount of variance as all participants experience having the condition. High *R*^2^ values are important in models designed for clinical prediction, but much less so when models are used for testing hypotheses about possible associations of variables of interest with critical patient-important outcomes. In this case, including in the present study, having a sufficiently large sample size to generate reasonably precise parameter estimates is a more important consideration [29].

People with SSc experience multiple challenges, including mental health issues such as depression and anxiety symptoms, as well as other physical symptoms such as pain, fatigue and itch, and concerns such as fear of disease progression [4]. Depression and anxiety screening has been recommended by some in RA, psoriasis and PsA [37, 38]. However, no trials have evaluated whether screening would improve mental health outcomes in any rheumatology settings. Randomized controlled trials have evaluated the effects of screening for depression in postpartum women, patients with osteoarthritis, patients with post-acute coronary syndrome, and post-deployment military personnel, and none have found that depression screening improved mental health outcomes [39]; to date, there are no trials of screening for anxiety disorders [40]. Mental health screening would require referral of large numbers of patients for psychiatric assessment, and some patients would be treated. But, based on trials conducted in other medical conditions, this would not likely improve mental health.

Thus, clinicians who care for people with SSc are encouraged to engage patients in discussions about their overall well-being, including mental health, and recognize that evaluating anxiety, if there is concern, may be a process that takes more than a single consultation [39]. Patient-centered approaches to ask appropriate questions and following up with assessments or referral for assessment are important. Providing information on self-help programs or peer support resources that may be available can be a first step in providing psychological support [10, 39].

Our study is the first large study of factors associated with symptoms of anxiety in individuals with SSc, with a sample size many times larger than the only other existing study of its kind. Other notable strengths include the use of a large international cohort with participants from over 47 sites across seven countries. There are also limitations. First, the SPIN Cohort is a convenience sample, and participants need to answer questionnaires online, both of which may reduce generalizability. Because of the convenience sampling, it is not possible to compare to patients at SPIN sites who would have been eligible but were not recruited or did not accept to participate. A comparison with the European Scleroderma Trials and Research and Canadian Scleroderma Research Group cohorts, however, found that participants were similar, which supports generalizability of SPIN Cohort results in SSc [15]. Second, the study assessed symptoms of anxiety and not anxiety disorders as assessed by diagnostic criteria. We compared PROMIS-29 v2.0 scale anxiety scores to the PROMIS normative reference population, which was based on the United States general population but is the only normative data for the PROMIS-29 Anxiety domain. Our sample included participants from multiple countries and had more females, older participants, and people who reported non-White ethnicity than the reference population. Third, this study was a cross-sectional study, thus causality cannot be determined. Instead, the study can only identify factors that may be plausibly causally related to anxiety. Fourth, some estimated associations, such as with primary biliary cholangitis, were large but imprecise due to large confidence intervals because the variable was present in few participants. This limits the ability to draw conclusions about such findings. Fifth, some disease variables in the SPIN Cohort are measured dichotomously without information on severity. This imperfect measurement may explain why, prior to including an interaction term, we found that having interstitial lung disease was associated with less anxiety. Imperfect measurement of model variables can lead to biased or spurious associations [41, 42]. In our study, imperfect measurement of interstitial lung disease may have resulted in confounding with country in our initial model.

Our results suggest that although many factors are associated with symptoms of anxiety in individuals with SSc, there are no single robust predictors to identify people with anxiety symptoms. We identified many different sociodemographic variables associated with greater anxiety, including female sex, self-reported race or ethnicity other than White, plus variables that reflected worse disease, particularly gastrointestinal involvement. More research is needed to better understand patterns of anxiety and potential causes and to develop interventions to target anxiety symptoms sources and support coping. Health-care providers should work with patients to identify and address sociodemographic and SSc-related characteristics that are associated with anxiety symptoms.

## Supplementary Material

keaf374_Supplementary_Data

## Data Availability

The data underlying this article will be shared on reasonable request to the corresponding author.
