# Supplementary material for: Anxiety symptoms and associated factors in the Scleroderma Patient-centered Intervention Network (SPIN) cohort: a cross-sectional study
Source: Rheumatology (Oxford). 2025 Aug 13;64(12):6268–77. doi: 10.1093/rheumatology/keaf374 (PMC12671864; doi:10.1093/rheumatology/keaf374)
Supplement: keaf374_Supplementary_Data [file keaf374_supplementary_data.docx]

**Supplementary: Table of Contents**

**Supplementary Data S1:** Medical variable data collection form and participant demographic form (pp 2-8)

**Supplementary Table S1:** Variable specifications (pp 9-11)

**Supplementary Table S2.** Linear regression analysis of sociodemographic and disease characteristic associations with anxiety symptoms (post-hoc analysis adding country x interstitial lung disease interaction) (p 12)

**Supplementary Table S3:** Linear regression analysis of sociodemographic and disease characteristic associations with anxiety symptoms (complete case analysis; N = 1,736) (p 13)

**Supplementary Table S4:** Linear regression analysis of sociodemographic and disease characteristic associations with anxiety symptoms (sensitivity analysis; N = 2,463; adding pruritus and pain intensity) (p 14)

**Supplementary Table S5:** Linear regression analysis of sociodemographic and disease characteristic associations with anxiety symptoms (sensitivity analysis; N = 2,463; replacing disease subtype with continuous Modified Rodnan Skin Scores) (p 15)

**Supplementary Table S6:** Linear regression analysis of sociodemographic and disease characteristic associations with anxiety symptoms (sensitivity analysis; N = 2,463; adding SSc-related antibodies) (p 16)

**SPIN Investigators** (p 17)

**Supplementary Data S1:** Medical variable data collection form, participant demographic form, and pruritus and itch items. the PROMIS-29v2.0 Anxiety domain items cannot be provided due to copyright restrictions

**Medical Variable Data Collection Form**

**Participant Demographic Form**

**Supplementary Table S1.** Variable specifications

| **Category** | **Variable** | **Regression model** | **Analyses** |
| --- | --- | --- | --- |
| Sociodemographic characteristics |  |  |  |
|  | Age, years | Continuous | All analyses |
|  | Sex | Dichotomous; Reference: female (versus. male) | All analyses |
|  | Education, years | Continuous | All analyses |
|  | Race/ethnicity | Dichotomous; Reference: White (versus. non-White) | All analyses |
|  | Marital status | Dichotomous; Reference: married or living as married (versus. single, divorced/separated, widowed) | All analyses |
|  | Country | Categorical; Reference United States (versus. Canada, United Kingdom, France, other (Australia, Mexico, Spain)) | All analyses |
| Lifestyle and BMI |  |  |  |
|  | Alcohol consumption, drinks/week | Continuous | Imputation analysis |
|  | Smoking status | Dichotomous; Reference: non-smoker (versus. current smoker) | Imputation analysis |
|  | PROMIS Variables: depression, sleep, fatigue, physical function, social functioning | Continuous | Imputation analysis |
|  | Body mass index, BMI score | Continuous | All analyses |
| Disease characteristics |  |  |  |
|  | Disease subtype | Binary; Reference: limited or sine SSc (versus. diffuse SSc) | Main analysis |
|  | Time since first non-Raynaud’s symptom, years | Continuous | All analyses |
|  |  |  |  |
|  | Modified Rodnan skin score | Continuous | Imputation analysis (replacing disease subtype) |
|  | Gastrointestinal involvement | Dichotomous; Reference: no GI involvement (versus upper GI involvement (esophagus) and/or lower GI involvement (stomach and/or intestines)) | All analyses |
|  | Digital ulcers | Binary; Reference: none (versus. anywhere on the finger) | All analyses |
|  | Current tendon friction rubs | Categorical; Reference: never present present (versus. currently present, present in the past) | All analyses |
|  | Small joint contractures | Categorical; Reference: none or mild contractures (versus. moderate contractures, severe contractures) | All analyses |
|  | Large joint contractures | Categorical; Reference: none or mild contractures (versus. moderate contractures, severe contractures) | All analyses |
|  | History of SSc Renal Crisis | Dichotomous; reference: no (versus. yes) | All analyses |
|  | Interstitial lung disease | Dichotomous; reference: no (versus. yes) | All analyses |
|  | Pulmonary arterial hypertension | Dichotomous; reference: no (versus. yes) | All analyses |
|  | Severity of itch in the last 7 days (0-11) | Continuous | Sensitivity analysis |
|  | Pain intensity | Continuous | Sensitivity analysis |
|  | Pain interference | Continuous | Imputation analysis |
| Presence of overlap syndromes | Systemic lupus erythematosus | Dichotomous; reference: no (versus. yes) | All analyses |
|  | Rheumatoid arthritis | Dichotomous; reference: no (versus. yes) | All analyses |
|  | Sjogren’s syndrome | Dichotomous; reference: no (versus. yes) | All analyses |
|  | Autoimmune thyroid disease | Dichotomous; reference: no (versus. yes) | All analyses |
|  | Idiopathic inflammatory myositis | Dichotomous; reference: no (versus. yes) | All analyses |
|  | Primary biliary cirrhosis | Dichotomous; reference: no (versus. yes) | All analyses |
| Antibodies | Anti-nuclear antibodies | Dichotomous; Reference: negative (versus. positive) | Sensitivity analysis |
|  | Anti-Centromere | Dichotomous; Reference: negative (versus. positive) | Sensitivity analysis |
|  | Anti-topoisomerase I [Scl70] | Dichotomous; Reference: negative (versus. positive) | Sensitivity analysis |
|  | Anti-RNA polymerase III | Dichotomous; Reference: negative (versus. positive) | Sensitivity analysis |

**Supplementary Table S2.** Linear regression analysis of sociodemographic and disease characteristic associations with anxiety symptoms (post-hoc analysis adding country x interstitial lung disease interaction)

|  | **Anxiety symptoms** |
| --- | --- |
|  | **Multivariable Regression Coefficient (95% CI)^a^** |
| Sociodemographic variables and body mass index (BMI) |  |
| Age (years) | **-0.11 (-0.14; -0.07)** |
| Male sex (reference = female) | **-1.84 (-3.02; -0.66)** |
| Years of education (years) | -0.09 (-0.20; 0.02) |
| Single, divorced/separated, or widowed (reference = married or living as married) | **0.99 (0.13; 1.84)** |
| Non-White (reference = White) | **1.80 (0.74; 2.86)** |
| Country (reference = United States) |  |
| Canada | **2.12 (0.78; 3.46)** |
| United Kingdom | **2.34 (0.65; 4.02)** |
| France | **2.93 (1.64; 4.21)** |
| Other (Australia, Mexico, Spain) | 0.14 (-2.53; 2.56) |
| BMI | **0.10 (0.03; 0.17)** |
| Disease variables |  |
| Years since first non-Raynaud’s symptoms (years) | **-0.08 (-0.13; -0.03)** |
| Diffuse subtype (reference = limited or sine) | 0.17 (-0.76; 1.10) |
| Gastrointestinal involvement | **2.68 (1.51; 3.86)** |
| Digital ulcers (reference = no) | 0.89 (-0.26; 2.03) |
| Tendon friction rubs (reference = never) |  |
| Current | 0.18 (-1.13; 1.48) |
| Past | -0.10 (-1.45; 1.24) |
| Small joint contractures (reference = none or mild) |  |
| Moderate | **1.28 (0.14; 2.42)** |
| Severe | 0.90 (-0.81; 2.60) |
| Large joint contractures (reference = none or mild) |  |
| Moderate | -0.03 (-1.51; 1.45) |
| Severe | 1.83 (-0.53; 4.19) |
| History of SSc renal crisis (reference = no) | 0.66 (-1.29; 2.60) |
| Interstitial lung disease (ILD; reference = no) | **0.21 (-1.17; 1.59)** |
| Pulmonary arterial hypertension (reference = no) | 0.17 (-1.25; 1.58) |
| Overlap syndromes |  |
| Systemic lupus erythematosus (reference = no) | -0.65 (-3.05; 1.74) |
| Rheumatoid arthritis (reference = no) | 0.90 (-0.84; 2.64) |
| Sjogren’s syndrome (reference = no) | **1.68 (0.17; 3.18)** |
| Autoimmune thyroid disease (reference = no) | -0.57 (-2.18; 1.03) |
| Idiopathic inflammatory myositis (reference = no) | -0.28 (-2.07; 1.52) |
| Primary biliary cirrhosis (reference = no) | 2.32 (-0.53; 5.17) |
| Country*ILD |  |
| Canada*ILD | -1.01 (-3.27; 1.25) |
| United Kingdom* ILD | -2.75 (-6.00; 0.50) |
| France* ILD | **-2.38 (-4.36; -0.39)** |
| Other (Australia, Mexico, Spain)* ILD | 0.21 (-4.03; 4.44) |

SSc = systemic sclerosis. Adjusted R^2^ = 0.06

^a^All regression coefficients are unstandardized. Bolded results were statistically significant.

**Supplementary Table S3.** Linear regression analysis of sociodemographic and disease characteristic associations with anxiety symptoms (complete case analysis; N = 1,736)

|  | **Anxiety symptoms** |
| --- | --- |
|  | **Multivariable Regression Coefficient (95% CI)^a^** |
| Sociodemographic variables and body mass index (BMI) |  |
| Age (years) | **-0.12 (-0.16; -0.08)** |
| Male sex (reference = female) | -1.03 (-2.44; 0.39) |
| Years of education (years) | -0.11 (-0.23; 0.02) |
| Single, divorced/separated, or widowed (reference = married or living as married) | **1.16 (0.15; 2.17)** |
| Non-White (reference = White) | **1.66 (0.41; 2.91)** |
| Country (reference = United States) |  |
| Canada | 1.33 (-0.01; 2.67) |
| United Kingdom | 0.65 (-1.31; 2.61) |
| France | **1.71 (0.50; 2.92)** |
| Other (Australia, Mexico, Spain) | -0.37 (-2.54; 1.80) |
| BMI | **0.11 (0.03; 0.19)** |
| Disease variables |  |
| Years since first non-Raynaud’s symptoms (years) | **-0.09 (-0.15; -0.04)** |
| Diffuse subtype (reference = limited or sine) | -0.19 (-1.30; 0.91) |
| Gastrointestinal involvement | **2.68 (1.26; 4.09)** |
| Digital ulcers (reference = no) | 0.64 (-0.79; 2.07) |
| Tendon friction rubs (reference = never) |  |
| Current | -0.19 (-1.69; 1.32) |
| Past | -0.16 (-1.84; 1.52) |
| Small joint contractures (reference = none or mild) |  |
| Moderate | **1.54 (0.14; 2.94)** |
| Severe | 0.83 (-1.26; 2.93) |
| Large joint contractures (reference = none or mild) |  |
| Moderate | 0.18 (-1.67; 2.04) |
| Severe | 1.39 (-1.86; 4.65) |
| History of SSc renal crisis (reference = no) | -0.47 (-2.99; 2.04) |
| Interstitial lung disease (reference = no) | **-1.07 (-2.09; -0.06)** |
| Pulmonary arterial hypertension (reference = no) | 0.50 (-1.27; 2.27) |
| Overlap syndromes |  |
| Systemic lupus erythematosus (reference = no) | 0.83 (-2.09; 3.74) |
| Rheumatoid arthritis (reference = no) | 0.90 (-1.44; 3.23) |
| Sjogren’s syndrome (reference = no) | 1.39 (-0.39; 3.17) |
| Autoimmune thyroid disease (reference = no) | 0.48 (-1.36; 2.31) |
| Idiopathic inflammatory myositis (reference = no) | 0.25 (-1.88; 2.37) |
| Primary biliary cirrhosis (reference = no) | 2.93 (-0.68; 6.55) |

Adjusted R^2^ = 0.06

^a^All regression coefficients are unstandardized. Bolded results were statistically significant.

**Supplementary Table S4.** Linear regression analysis of sociodemographic and disease characteristic associations with anxiety symptoms (sensitivity analysis; N = 2,463; adding pruritus and pain intensity)

|  | **Anxiety symptoms** |
| --- | --- |
|  | **Multivariable Regression Coefficient (95% CI)^a^** |
| Sociodemographic variables and body mass index (BMI) |  |
| Age (years) | **-0.10 (-0.13; -0.07)** |
| Male sex (reference = female) | -0.93 (-2.02; 0.16) |
| Years of education (years) | 0.04 (-0.06; 0.14) |
| Single, divorced/separated, or widowed (reference = married or living as married) | 0.56 (-0.23; 1.34) |
| Non-White (reference = White) | **1.01 (0.03; 1.99)** |
| Country (reference = United States) |  |
| Canada | **1.24 (0.24; 2.24)** |
| United Kingdom | 0.70 (-0.65; 2.05) |
| France | **1.69 (0.74; 2.64)** |
| Other (Australia, Mexico, Spain) | 0.83 (-1.07; 2.72) |
| BMI | 0.01 (-0.05; 0.08) |
| Disease variables |  |
| Years since first non-Raynaud’s symptoms (years) | **-0.08 (-0.13; -0.04)** |
| Diffuse subtype (reference = limited or sine) | -0.03 (-0.89; 0.83) |
| Gastrointestinal involvement | **1.72 (0.63; 2.80)** |
| Digital ulcers (reference = no) | 0.11 (-0.94; 1.17) |
| Tendon friction rubs (reference = never) |  |
| Current | -0.32 (-1.52; 0.88) |
| Past | -0.26 (-1.50; 0.98) |
| Small joint contractures (reference = none or mild) |  |
| Moderate | 0.27 (-0.78; 1.32) |
| Severe | 0.38 (-1.19; 1.95) |
| Large joint contractures (reference = none or mild) |  |
| Moderate | -0.93 (-2.30; 0.44) |
| Severe | 0.82 (-1.35; 3.00) |
| History of SSc renal crisis (reference = no) | 1.07 (-0.72; 2.87) |
| Interstitial lung disease (reference = no) | -0.76 (-1.55; 0.03) |
| Pulmonary arterial hypertension (reference = no) | 0.19 (-1.12; 1.49) |
| Pruritus | **0.27 (0.12; 0.42)** |
| Pain intensity | **1.41 (1.25; 1.57)** |
| Overlap syndromes |  |
| Systemic lupus erythematosus (reference = no) | -0.59 (-2.78; 1.61) |
| Rheumatoid arthritis (reference = no) | -0.18 (-1.78; 1.43) |
| Sjogren’s syndrome (reference = no) | 1.14 (-0.24; 2.52) |
| Autoimmune thyroid disease (reference = no) | -0.34 (-1.81; 1.14) |
| Idiopathic inflammatory myositis (reference = no) | -0.67 (-2.32; 0.97) |
| Primary biliary cirrhosis (reference = no) | 2.22 (-0.41; 4.84) |

Adjusted R^2^ = 0.20

^a^All regression coefficients are unstandardized. Bolded results were statistically significant

**Supplementary Table S5.** Linear regression analysis of sociodemographic and disease characteristic associations with anxiety symptoms (sensitivity analysis; N = 2,463; replacing disease subtype with continuous Modified Rodnan Skin Scores)

|  | **Anxiety symptoms** |
| --- | --- |
|  | **Multivariable Regression Coefficient (95% CI)^a^** |
| Sociodemographic variables and body mass index (BMI) |  |
| Age (years) | **-0.11 (-0.14; -0.07)** |
| Male sex (reference = female) | **-1.80 (-2.98; -0.62)** |
| Years of education (years) | -0.09 (-0.20; 0.02) |
| Single, divorced/separated, or widowed (reference = married or living as married) | **0.99 (0.14; 1.84)** |
| Non-White (reference = White) | **1.75 (0.69; 2.81)** |
| Country (reference = United States) |  |
| Canada | **1.69 (0.60; 2.78)** |
| United Kingdom | **1.54 (0.07; 3.00)** |
| France | **2.05 (1.03; 3.07)** |
| Other (Australia, Mexico, Spain) | 0.04 (-2.01; 2.09) |
| BMI | **0.10 (0.03; 0.17)** |
| Disease variables |  |
| Years since first non-Raynaud’s symptoms (years) | **-0.08 (-0.12; -0.03)** |
| Modified Rodnan Skin Score | 0.03 (-0.03; 0.09) |
| Gastrointestinal involvement | **2.69 (1.52; 3.87)** |
| Digital ulcers (reference = no) | 0.81 (-0.35; 1.96) |
| Tendon friction rubs (reference = never) |  |
| Current | 0.10 (-1.21; 1.41) |
| Past | -0.13 (-1.45; 1.20) |
| Small joint contractures (reference = none or mild) |  |
| Moderate | 1.13 (-0.02; 2.27) |
| Severe | 0.67 (-1.06; 2.39) |
| Large joint contractures (reference = none or mild) |  |
| Moderate | -0.10 (-1.59; 1.39) |
| Severe | 1.72 (-0.65; 4.09) |
| History of SSc renal crisis (reference = no) | 0.72 (-1.22; 2.65) |
| Interstitial lung disease (reference = no) | **-0.98 (-1.84; -0.13)** |
| Pulmonary arterial hypertension (reference = no) | 0.15 (-1.26; 1.56) |
| Overlap syndromes |  |
| Systemic lupus erythematosus (reference = no) | -0.69 (-3.07; 1.70) |
| Rheumatoid arthritis (reference = no) | 0.96 (-0.78; 2.70) |
| Sjogren’s syndrome (reference = no) | **1.72 (0.22; 4.22)** |
| Autoimmune thyroid disease (reference = no) | -0.59 (-2.19; 1.01) |
| Idiopathic inflammatory myositis (reference = no) | -0.43 (-2.22; 1.35) |
| Primary biliary cirrhosis (reference = no) | 2.38 (-0.47; 5.23) |

Adjusted R^2^ = 0.06

^a^All regression coefficients are unstandardized. Bolded results were statistically significant

.

**Supplementary Table S6.** Linear regression analysis of sociodemographic and disease characteristic associations with anxiety symptoms (sensitivity analysis; N = 2,463; adding SSc-related antibodies)

|  | **Anxiety symptoms** |
| --- | --- |
|  | **Multivariable Regression Coefficient (95% CI)^a^** |
| Sociodemographic variables and body mass index (BMI) |  |
| Age (years) | **-0.11 (-0.14; -0.07)** |
| Male sex (reference = female) | **-1.89 (-3.08; -0.70)** |
| Years of education (years) | -0.09 (-0.20; 0.02) |
| Single, divorced/separated, or widowed (reference = married or living as married) | **1.01 (0.16; 1.86)** |
| Non-White (reference = White) | **1.79 (0.73; 2.85)** |
| Country (reference = United States) |  |
| Canada | **1.74 (0.65; 2.83)** |
| United Kingdom | **1.54 (0.08; 3.01)** |
| France | **2.04 (1.01; 3.08)** |
| Other (Australia, Mexico, Spain) | 0.05 (-2.01; 2.11) |
| BMI | **0.10 (0.03; 0.17)** |
| Disease variables |  |
| Years since first non-Raynaud’s symptoms (years) | **-0.08 (-0.13; -0.04)** |
| Diffuse subtype (reference = limited or sine) | 0.15 (-0.84; 1.15) |
| Gastrointestinal involvement | **2.76 (1.58; 3.94)** |
| Digital ulcers (reference = no) | 1.04 (-0.12; 2.19) |
| Tendon friction rubs (reference = never) |  |
| Current | 0.24 (-1.07; 1.54) |
| Past | 0.01 (-1.34; 1.36) |
| Small joint contractures (reference = none or mild) |  |
| Moderate | **1.29 (0.14; 2.43)** |
| Severe | 0.93 (-0.78; 2.65) |
| Large joint contractures (reference = none or mild) |  |
| Moderate | -0.00 (-1.49; 1.49) |
| Severe | 1.80 (-0.57; 4.16) |
| History of SSc renal crisis (reference = no) | 0.95 (-1.03; 2.93) |
| Interstitial lung disease (reference = no) | **-0.97 (-1.91; -0.03)** |
| Pulmonary arterial hypertension (reference = no) | 0.16 (-1.26; 1.58) |
| Overlap syndromes |  |
| Systemic lupus erythematosus (reference = no) | -0.78 (-3.17; 1.62) |
| Rheumatoid arthritis (reference = no) | 0.88 (-0.86; 2.63) |
| Sjogren’s syndrome (reference = no) | **1.71 (0.20; 3.21)** |
| Autoimmune thyroid disease (reference = no) | -0.51 (-2.11; 1.10) |
| Idiopathic inflammatory myositis (reference = no) | -0.67 (-2.47; 1.13) |
| Primary biliary cirrhosis (reference = no) | 2.40 (-0.47; 5.27) |
| SSc-related antibodies |  |
| Antinuclear antibodies (reference = negative) | -0.07 (-1.82; 1.68) |
| Anti-centromere (reference = negative) | -0.60 (-1.63; 0.43) |
| Anti-topoisomerase I [Scl70] (reference = negative) | -0.80 (-1.83; 0.22) |
| Anti-RNA polymerate III (reference = negative) | -0.86 (-2.06; 0.34) |

Adjusted R^2^ = 0.06

^a^All regression coefficients are unstandardized. Bolded results were statistically significant

**SPIN Investigators**

Claire Elizabeth Adams, Jewish General Hospital, Montreal, Quebec, Canada; Christian Agard, Centre Hospitalier Universitaire - Hôtel-Dieu de Nantes, Nantes, France; Laurent Alric, CHU Rangueil, Toulouse, France; Marc André, Centre Hospitalier Universitaire Gabriel-Montpied, Clermont-Ferrand, France; Floryan Beaslay, CHU La Réunion, Saint-Denis, La Réunion, France; Elana J. Bernstein, Columbia University, New York, New York, USA; Sabine Berthier, Centre Hospitalier Universitaire Dijon Bourgogne, Dijon, France; Lyne Bissonnette, Université de Sherbrooke, Sherbrooke, Quebec, Canada; Sophie Blaise, CHU Grenoble Alpes, Grenoble, France; Eva Bories, CHU Rangueil, Toulouse, France; Alessandra Bruns, Université de Sherbrooke, Sherbrooke, Quebec, Canada; Carlotta Cacciatore, Assistance Publique – Hôpitaux de Paris, Hôpital St-Louis, Paris, France; Patricia Carreira, Servicio de Reumatologia del Hospital 12 de Octubre, Madrid, Spain; Marion Casadevall, Assistance Publique - Hôpitaux de Paris, Hôpital Cochin, Paris, France; Benjamin Chaigne, Assistance Publique - Hôpitaux de Paris, Hôpital Cochin, Paris, France; Lorinda Chung, Stanford University, Stanford, California, USA; Benjamin Crichi, Assistance Publique - Hôpitaux de Paris, Hôpital St-Louis, Paris, France; Thylbert Deltombe, CHU La Réunion, Saint-Denis, La Réunion, France; Christopher P. Denton, Royal Free London Hospital, London, UK; Tannvir Desroche, CHU La Réunion, Saint-Denis, La Réunion, France; Robyn Domsic, University of Pittsburgh, Pittsburgh, Pennsylvania, USA; James V. Dunne, St. Paul's Hospital and University of British Columbia, Vancouver, British Columbia, Canada; Bertrand Dunogue, Assistance Publique - Hôpitaux de Paris, Hôpital Cochin, Paris, France; Regina Fare, Servicio de Reumatologia del Hospital 12 de Octubre, Madrid, Spain; Dominique Farge-Bancel, Assistance Publique - Hôpitaux de Paris, Hôpital St-Louis, Paris, France; Paul R. Fortin, CHU de Québec - Université Laval, Quebec, Quebec, Canada; Tracy Frech, Vanderbilt University, Nashville, Tennessee, USA; Loraine Gauzère, CHU La Réunion, Saint-Denis, La Réunion, France; Anne Gerber, CHU La Réunion, Saint-Denis, La Réunion, France; Jessica K. Gordon, Hospital for Special Surgery, New York City, New York, USA; Brigitte Granel-Rey, Université, and Assistance Publique - Hôpitaux de Marseille, Hôpital Nord, Marseille, France; Aurélien Guffroy, Les Hôpitaux Universitaires de Strasbourg, Nouvel Hôpital Civil, Strasbourg, France; Geneviève Gyger, Jewish General Hospital and McGill University, Montreal, Quebec, Canada; Eric Hachulla, Centre Hospitalier Régional Universitaire de Lille, Hôpital Claude Huriez, Lille, France; Daphna Harel, New York University, NewYork, New York, USA; Monique Hinchcliff, Yale School of Medicine, New Haven, Connecticut, USA; Sabrina Hoa, Centre hospitalier de l’Université de Montréal – CHUM, Montreal, Quebec, Canada; Michael Hugues, Salford Royal NHS Foundation Trust, Salford, UK; Alena Ikic, CHU de Québec - Université Laval, Quebec, Quebec; Sindhu R. Johnson, Toronto Scleroderma Program, Mount Sinai Hospital, Toronto Western Hospital, and University of Toronto, Toronto, Ontario, Canada; Nader Khalidi, McMaster University, Hamilton, Ontario, Canada; Kimberly S. Lakin, Hospital for Special Surgery, New York City, New York, USA; Marc Lambert, Centre Hospitalier Régional Universitaire de Lille, Hôpital Claude Huriez, Lille, France; Maggie Larche, University of Calgary, Calgary, Alberta, Canada; David Launay, Centre Hospitalier Régional Universitaire de Lille, Hôpital Claude Huriez, Lille, France; Yvonne C. Lee, Northwestern University, Chicago, Illinois, USA; Paul Legendre, Centre Hospitalier du Mans, Le Mans, France; Catarina Leite, University of Minho, Braga, Portugal; Hélène Maillard, Centre Hospitalier Régional Universitaire de Lille, Hôpital Claude Huriez, Lille, France; Nancy Maltez, University of Ottawa, Ottawa, Ontario, Canada; Joanne Manning, Salford Royal NHS Foundation Trust, Salford, UK; Isabelle Marie, CHU Rouen, Hôpital de Bois-Guillaume, Rouen, France; Maria Martin Lopez, Servicio de Reumatologia del Hospital 12 de Octubre, Madrid, Spain; Thierry Martin, Les Hôpitaux Universitaires de Strasbourg, Nouvel Hôpital Civil, Strasbourg, France; Ariel Masetto, Université de Sherbrooke, Sherbrooke, Quebec, Canada; Arsène Mekinian, Assistance Publique - Hôpitaux de Paris, Hôpital St-Antoine, Paris, France; Sheila Melchor Díaz, Servicio de Reumatologia del Hospital 12 de Octubre, Madrid, Spain; Morgane Mourguet, CHU Rangueil, Toulouse, France; Christelle Nguyen, Université Paris Descartes, Université de Paris, Paris, France, and Assistance Publique - Hôpitaux de Paris, Paris, France; Karen Nielsen, Scleroderma Society of Ontario, Hamilton, Ontario, Canada; Mandana Nikpour, St Vincent’s Hospital and University of Melbourne, Melbourne, Victoria, Australia; Louis Olagne, Centre Hospitalier Universitaire Gabriel-Montpied, Clermont-Ferrand, France; Vincent Poindron, Les Hôpitaux Universitaires de Strasbourg, Nouvel Hôpital Civil, Strasbourg, France; Janet Pope, University of Western Ontario, London, Ontario, Canada; Susanna Proudman, Royal Adelaide Hospital and University of Adelaide, Adelaide, South Australia, Australia; Grégory Pugnet, CHU Rangueil, Toulouse, France; Loïc Raffray, CHU La Réunion, Saint-Denis, La Réunion, France; François Rannou, Université Paris Descartes, Université de Paris, Paris, France, and Assistance Publique - Hôpitaux de Paris, Paris, France; Alexis Régent, Assistance Publique - Hôpitaux de Paris, Hôpital Cochin, Paris, France; Frederic Renou, CHU La Réunion, Saint-Denis, La Réunion, France; Sébastien Rivière, Assistance Publique - Hôpitaux de Paris, Hôpital St-Antoine, Paris, France; David Robinson, University of Manitoba, Winnipeg, Manitoba, Canada; Esther Rodríguez Almazar, Servicio de Reumatologia del Hospital 12 de Octubre, Madrid, Spain; Tatiana Sofia Rodríguez-Reyna, Instituto Nacional de Ciencias Médicas y Nutrición Salvador Zubirán, Mexico City, Mexico; Sophie Roux, Université de Sherbrooke, Sherbrooke, Quebec, Canada; Perrine Smets, Centre Hospitalier Universitaire Gabriel-Montpied, Clermont-Ferrand, France; Vincent Sobanski, Centre Hospitalier Régional Universitaire de Lille, Hôpital Claude Huriez, Lille, France; Robert F. Spiera, Hospital for Special Surgery, New York City, New York, USA; Virginia Steen, Georgetown University, Washington, DC, USA; Evelyn Sutton, Dalhousie University, Halifax, Nova Scotia, Canada; Carter Thorne, Southlake Regional Health Centre, Newmarket, Ontario, Canada; Damien Vagner, CHU La Réunion, Saint-Denis, La Réunion, France; John Varga, University of Michigan, Ann Arbor, Michigan, USA; Pearce Wilcox, St. Paul's Hospital and University of British Columbia, Vancouver, British Columbia, Canada; Mara Cañedo Ayala, Jewish General Hospital, Montreal, Quebec, Canada; Vanessa Cook, Jewish General Hospital, Montreal, Quebec, Canada; Cassidy Dal Santo, Jewish General Hospital, Montreal, Quebec; Tiffany Dal Santo, Jewish General Hospital, Montreal, Quebec; Monica D’Onofrio, Jewish General Hospital, Montreal, Quebec; Sophie Hu, Jewish General Hospital, Montreal, Quebec, Canada; Elsa-Lynn Nassar, Jewish General Hospital, Montreal, Quebec, Canada; Marieke Alexandra Neyer, Jewish General Hospital, Montreal, Quebec, Canada.
